# Association of Nightly Fasting, Meal Frequency, and Skipping Meals with Metabolic Syndrome among Kuwaiti Adults

**DOI:** 10.3390/nu16070984

**Published:** 2024-03-28

**Authors:** Fatema Alkhulaifi, Suad Al-Hooti, Sameer Al-Zenki, Husam Alomirah, Qian Xiao, Wenyaw Chan, Fuqing Wu, Charles Darkoh

**Affiliations:** 1 Department of Epidemiology, Human Genetics & Environmental Sciences, School of Public Health, University of Texas Health Science Center at Houston, Houston, TX 77030, USA; fatema.alkhulaifi@uth.tmc.edu (F.A.); qian.xiao@uth.tmc.edu (Q.X.); wenyaw.chan@uth.tmc.edu (W.C.); fuqing.wu@uth.tmc.edu (F.W.); 2 Department of Epidemiology and Biostatistics, College of Public Health, Kuwait University, Khaldiya 12037, Kuwait; 3 Kuwait Institute of Scientific Research, Kuwait City 13109, Kuwait; suad.alhooti@gmail.com (S.A.-H.); szenki@kisr.edu.kw (S.A.-Z.); dralomirah@gmail.com (H.A.); 4 Microbiology and Infectious Diseases Program, Graduate School of Biomedical Sciences, University of Texas MD Anderson Cancer Center UTHealth, Houston, TX 77030, USA

**Keywords:** meal frequency, nightly fasting, skipping meals, late night dinner, metabolic syndrome, Kuwait

## Abstract

Mounting evidence suggests that meal timing and frequency are associated with cardiometabolic health by influencing circadian rhythms. However, the evidence is inconsistent and limited, especially in non-Western cultures. This cross-sectional study aims to investigate the association between temporal habits of dietary intake, such as nightly fasting duration and meal frequency, and metabolic syndrome among Kuwaiti adults. A 24-hour recall was used to assess temporal habits of dietary intake. Meal frequency was defined as the number of daily eating episodes. The study included a total of 757 adults aged 20 years and older. The participants’ mean age was 37.8 ± 12.3 years. After adjusting for all confounders, higher meal frequency was found to be associated with a lower prevalence of metabolic syndrome in adults (OR, 0.43; 95%CI, 0.19–0.96) and a lower prevalence of elevated triglycerides in men only (OR, 0.23; 95%CI, 0.09–0.60). No association was found between nightly fasting and metabolic syndrome, but a longer fasting duration was associated with a lower prevalence of elevated triglycerides (OR, 0.19; 95%CI, 0.06–0.63). The findings suggest that having frequent meals and longer durations of nightly fasting may help decrease the risk of metabolic syndrome and elevated triglycerides.

## 1. Introduction

It is estimated that 20–30% of adults worldwide have metabolic syndrome [1]. Along with the increased risk of coronary heart disease, stroke [2], and other serious health conditions, metabolic syndrome affects quality of life [3] and carries a heavy health expenditure [4]. Although many lifestyle factors have been associated with metabolic syndrome, the association between temporal patterns of dietary intake and metabolic syndrome has only been studied in recent years [5,6,7].

The temporal patterns of dietary intake (e.g., meal timing, meal frequency, breakfast skipping, and late-night eating) are gaining interest as compelling evidence shows that these are potentially important risk factors that may impact human health, especially metabolic-related diseases [6]. The circadian rhythm, the body’s inborn 24 h cycle that impacts behaviors and physiological processes, is essential in understanding how the body is affected by meal timings. As modern society developed, people became exposed more to a nocturnal lifestyle [8], which has led to increased exposure to artificial light and access to food any time of the day [9]. Environmental changes, temperature, exercise, and meal timings can affect the circadian rhythm and the body’s metabolic process [10]. Therefore, temporal patterns of dietary intake, such as meal timing and meal frequency, are suggested to influence weight control and cardiometabolic health [9,11].

There have been limited studies on the association between temporal patterns of dietary intake and cardiometabolic risk in humans. For the frequency of meals, an observational study has revealed that eating four meals a day was associated with a lower risk of developing type 2 diabetes when compared with eating three meals a day in Chinese adults, especially in those with a BMI of less than 25 kg/m^2^ [12]. Furthermore, a lower concentration of LDL cholesterol was found among participants in the EPIC project who consumed more than six meals per day as compared with those who consumed one or two meals per day [13]. As for fasting duration, a cross-sectional study of Korean adults found no significant linear association between nightly fasting duration and obesity or metabolic syndrome [7]. Another study revealed that a longer duration of night-time fasting does reduce the likelihood of being overweight and obese but only among older adults who started fasting before 2 a.m. [14]. The studies were mostly inconsistent and found relationships in one group but not in the other. Thus, there is not enough evidence to draw comprehensive conclusions about the associations between temporal and diurnal patterns of dietary intake and metabolic syndrome.

The rise in rates of metabolic syndrome is a global phenomenon. Most studies on the association of temporal patterns of dietary intake and metabolic syndrome are conducted in Western or Asian countries. The prevalence of metabolic syndrome increases as a country’s level of income increases [15]. Thus, Kuwait provides fertile grounds for the development of metabolic syndrome with the country’s high income, sedentary lifestyle [16], and eating culture. In Kuwait, the prevalence of metabolic syndrome is 37.7% in women and 34.2% in men [17], higher than the 20–25% worldwide estimate of metabolic syndrome in adults [18]. Many factors may be associated with the increased rate of metabolic syndrome in Kuwait, such as screen time and less night sleep. However, it is essential to explore the association between temporal patterns of dietary intake, such as meal timing and meal frequency, and metabolic syndrome. This study explored meal timing, meal frequency, fasting duration, and skipping meals, and examined their association with metabolic syndrome in Kuwaiti adults using national survey data.

## 2. Materials and Methods

### 2.1. The Study Participants

The participants of the study are adults aged 20 years and above who participated in the 2009–2010 Kuwait National Nutrition Surveillance System (KNNSS). The study collected a nationally representative sample by following a stratified cluster sampling strategy obtained from 545 individual households in Kuwait’s six governorates. The governorates were divided into 82 clusters to sample the households, where 20 households were selected from each cluster, aiming for a total of 1640 households. From each household, subjects were randomly selected. In total, 1824 individuals participated in the KNNSS study. For this study, 757 adults above 20 years old were included. Those under 20 years old, 722 individuals in total, were excluded because they were assessed differently than adults. The study excluded 345 individuals who were taking diabetes, blood pressure, or cholesterol medications.

This study was conducted in accordance with the guidelines laid down in the Declaration of Helsinki, and all procedures involving human subjects/patients were approved by the Ethics Committee of the Ministry of Health in Kuwait. Written informed consent was obtained from all subjects.

### 2.2. Assessment of Dietary Data

Participants completed a 24 h dietary recall as part of the KNNSS. All interviewers conducting the 24 h recall were KNNSS-trained dietitians. A food instruction booklet was used to aid participants in estimating portion size. The time a person ate a meal was defined as meal timing. The number of eating episodes was estimated as meal frequency. An eating episode was defined as consuming any food or drink of more than 1 kcal. Not eating a meal from 5 a.m. to 10 a.m. was defined as skipping breakfast. Not eating a meal from 6 p.m. to 10 p.m. was defined as skipping dinner. Eating a meal between 10 p.m. and 3 a.m. was defined as late-night eating. Fasting duration was estimated by subtracting the time of the last meal from the time of the first meal. Individuals were classified based on meal frequency into four categories: those who consumed three meals or less, four meals, five meals, and six meals or more. For fasting duration, individuals were classified based on nightly fasting duration into four categories: those who fast for less than 8 h, those who fast for 8 to 10 h, those who fast for 10 to 12 h, and those who fast for more than 12 h.

### 2.3. Covariate Assessment

Information related to demographic background, physical activity, and lifestyle factors was collected through interviewer-administered questionnaires. Trained workers visited households to collect body and blood measurements. Following standard protocol, two measurements were taken for body weight, height, blood pressure, and waist circumference. Blood samples were collected to measure biomarkers after a nightly fast. Body mass index (BMI) was calculated as weight in kilograms divided by height in square meters. Individuals were classified as being underweight if they had a BMI < 18.5, being at a normal weight if they had a BMI between 18.5 and <25, being overweight if they had a BMI between 25 and <30, and being obese if they had a BMI > 30. Age was categorized in the questionnaire into two categories: 20–49 years, and 50 years and older. Age was also collected as a continuous variable. Smoking was defined in terms of having ever smoked a cigarette (Yes/No). Physical activity was defined as being physically active or having a sedentary lifestyle (Active/Sedentary). Marital status was defined according to those who are married and those who are not married, including divorced and widowed (Yes/No). Education was classified into three categories (high school and less, diploma/bachelor’s, and postgraduate). Household income was defined as having a low (<1000 Kuwaiti dinar (KD)), medium (1000–1999 KD), or high income (>2000 KD). Total daily energy intake was estimated from the 24 h recall.

### 2.4. Assessment of Outcomes

Metabolic syndrome was defined as having three or more metabolic syndrome risk factors. The risk factors include elevated blood pressure, prediabetes or elevated fasting blood glucose, abdominal obesity or large waist circumference, elevated levels of triglycerides, and reduced levels of high-density lipoprotein (HDL). Abdominal obesity was defined as having a waist circumference ≥ 94 cm for men and ≥80 cm for women. Elevated blood pressure (BP) was defined as having a diastolic BP ≥ 80 mm hg and/or systolic BP ≥ 120 mm Hg. Prediabetes was defined as having a fasting blood glucose ≥ 100 mg/dL (5.6 mmol/L) or HbA1c ≥ 5.7. Having elevated triglycerides was defined as having serum triglycerides in amounts ≥ 150 mg/dL (1.7 mmol/L). Low HDL cholesterol was defined as having an HDL cholesterol level < 40 mg/dL (1.0 mmol/L) for men and <50 mg/dL (1.3 mmol/L) for women.

### 2.5. Statistical Analysis

Continuous variables are presented as means ± standard deviations, and categorical variables are presented as frequencies (*n*) and/or percentiles (%). A *t*-test was conducted to identify if there was a difference in characteristics between men and women. A Chi-square test was conducted for the difference in temporal patterns of dietary intake, metabolic syndrome risk factors, and the number of metabolic syndrome risk factors present between men and women. Analysis was conducted using the SURVEY procedure (SAS version 9.4) [19] to account for the sampling weights. Multiple logistic regression analyses were performed to estimate the odds ratios (OR) and the 95% confidence intervals (CI) of metabolic syndrome and risk factors of metabolic syndrome. For meal frequency, participants eating three or less meals per day were used as the reference group. For fasting duration, participants fasting less than 8 h were used as the reference group. The analysis was performed for all adults and then separately for men and women. Further multiple logistic regression analyses were performed separately based on breakfast consumption (skip breakfast; did not skip breakfast) and dinner consumption (skip dinner; did not skip dinner). The results are presented, adjusted for age in years, education, income, BMI, activity level, and smoking. A significance level of *p* < 0.05 was used for all statistical tests.

## 3. Results

Selected characteristics of the study participants are shown in Table 1. The mean age was 38.6 years for men and 37.1 years for women. The difference in age was not significant; however, there were significant differences in income (*p* < 0.001), smoking status (*p* < 0.001), HbA1c levels (*p* = 0.02), HDL cholesterol levels (*p* < 0.001), triglyceride levels (*p* < 0.001), and total energy intake (*p* < 0.001). Further, there was a significant difference in the activity level, in which 47.2% of men and 68.7% of women were active (*p* < 0.0001). On average, women had a slightly higher BMI (29.9 kg/m^2^) than did men (28.3 kg/m^2^) in the study (*p* = 0.0005). There was a significant difference between the waist circumference of men and women, in which men had a larger waist circumference (98.9 cm) than women (88.6). The mean systolic blood pressure was higher in men (129.1 mmHg) than in women (121.5 mmHg), and the difference was significant (*p* < 0.0001). Similar significant differences were in average diastolic blood pressure, in which the average for men was 82.7 mmHg and that for women was 78.6 mmHg. The mean fasting blood glucose level was 5.7 mmol/L in men and 5.4 mmol/L in women (*p* = 0.0245). On average, men consumed a statistically significantly higher number of meals (4.5 meals per day) than did women (4.3 meals per day) (*p* = 0.0005). Women had longer nightly fasts (10.9 h) than did men (10.1 h), and the difference was significant (*p* < 0.0001).

Furthermore, Table 1 shows the prevalence of metabolic syndrome and its five risk factors among the study participants. The prevalence of abdominal obesity was 40% for all study participants; however, there was a significant difference in the prevalence of abdominal obesity between men (61.0%) and women (63.0%). Further, men had a significantly higher prevalence of elevated serum triglycerides (29.9%) than did women (16.8%). On the other hand, the study showed a significantly higher prevalence of low HDL cholesterol among women (48.3%) than that in men (30.8%). The prevalence of elevated blood pressure was significantly higher among men (56.9%) than among women (37.7%). Approximately, 29.9% of the participants were prediabetic, and 34.5% had metabolic syndrome, in which no significant difference was seen between men and women. The number of participants without any, and with one or many of the risk factors associated with metabolic syndrome is illustrated in Table 1. Approximately, 12.6% of the participants had no metabolic syndrome risk factors present. The presence of one metabolic syndrome indicator was seen among 22.6% of the participants, with a significant difference between men (17.9%) and women (26.4%). Nearly 30% of participants had two metabolic syndrome risk factors present and 20% had three metabolic syndrome risk factors. Only 11.6% of participants had four metabolic syndrome risk factors, and 2.9% had all five risk factors present.

In the multiple logistic regression model (Table 2), adults in the highest quartile, with six meals or more in terms of meal frequency, had a lower prevalence of metabolic syndrome (OR, 0.43; 95%CI, 0.19–0.96) compared with adults who consume three meals or less daily. Furthermore, those consuming four meals per day had a lower prevalence of elevated blood pressure (OR, 0.54; 95%CI, 0.31–0.94) as compared with those who consume three meals or less per day. No other significant association between meal frequency and metabolic syndrome or metabolic syndrome risk factors was seen. For fasting duration, adults who fasted between 8 and 10 h daily had a lower prevalence of elevated triglycerides (OR, 0.47; 95%CI, 0.24–0.91) compared with adults fasting less than 8 h daily. Participants who fasted for longer periods, 10–12 h, had a higher prevalence of abdominal obesity (OR, 2.10; 95%CI, 1.00–4.44) compared with participants who fasted for 8 h or less. On the other hand, participants fasting more than 12 h daily had a higher prevalence of elevated blood pressure (OR, 1.91; 95%CI, 1.01–3.33) compared with those who fasted for 8 h or less daily. There was evidence of a significant linear trend of metabolic syndrome (*p* = 0.03), elevated blood pressure (*p* = 0.02), and elevated fasting blood glucose (*p* = 0.03) with nightly fasting duration.

Table 3 shows the multiple logistic regression analysis for men. Men who consume six meals or more per day had a lower prevalence of metabolic syndrome (OR, 0.26; 95%CI, 0.09–0.76) compared with men who ate three meals or less per day after controlling for age, income, education, BMI, activity level, and smoking. Moreover, after adjusting for the confounders, men consuming four meals daily had a lower prevalence of elevated triglycerides (OR, 0.31; 95%CI, 0.12–0.82) compared with men who consumed three meals or less daily. The prevalence was even lower (OR, 0.23; 95%CI, 0.09–0.60) for men who consumed five meals daily when compared with that for men who consumed three meals or less daily.

The multiple logistic regression analysis for women after adjusting for age, income, education, BMI, activity level, and smoking is shown in Table 4. Women eating four meals daily had a lower prevalence of elevated blood pressure (OR, 0.39; 95%CI, 0.18–0.86) when compared with women eating three meals or less daily after controlling for confounders. On the other hand, women who fasted for 10–12 h had a higher prevalence of elevated blood pressure (OR, 2.88; 95%CI, 1.15–7.20) when compared with women who fasted for less than 8 h after adjusting for confounders. Women who fasted for more than 12 h had a lower prevalence of elevated triglycerides (OR, 0.19; 95%CI, 0.06–0.63) when compared with women who fasted for less than 8 h after adjusting for confounders.

Table 5 shows the multiple logistic regression for those who skipped breakfast and those who did not skip breakfast for all adults after adjusting for age, gender, BMI, activity level, and smoking. Those who skipped breakfast and fasted nightly for more than 8 h had a lower prevalence of elevated triglycerides compared with those who fasted for less than 8 h. This association was true in all fasting categories, for those who had an 8 to 10 h fast (OR, 0.04; 95%CI, 0.006–0.25), 10 to 12 h fast (OR, 0.15; 95%CI, 0.03–0.90), and >12 h fast (OR, 0.14; 95%CI, 0.04–0.48). Adults who consumed breakfast and had a 10–12 h fast nightly had a higher prevalence of abdominal obesity (OR, 2.21; 95%CI, 1.04–4.71) than those who fasted for less than 8 h. Furthermore, adults who consumed breakfast and had a 10 to 12 h fast (OR, 2.23; 95%CI, 1.06–4.71) or more than 12 h fast (OR, 3.31; 95%CI, 1.27–8.59) had an increased prevalence of elevated triglycerides compared with those who fasted for less than 8 h. There was a linear relationship between fasting and high triglycerides among those consuming breakfast (*p* = 0.009).

Table 6 illustrates the multiple logistic regression for those who skipped dinner and those who did not skip dinner after adjusting for age, gender, BMI, activity level, and smoking. There is a lower prevalence of metabolic syndrome (OR, 0.06; 95%CI, 0.07–0.49) for those who skipped dinner and fasted for 8 to 10 h compared with those who fasted for less than 8 h. Moreover, fasting for 8 to 10 h was associated with a lower prevalence of abdominal obesity (OR, 0.02; 95%CI, 0.001–0.43) among those who skipped dinner compared with those who fasted for less than 8 h. On the other hand, among those who did not skip dinner, there was an increased prevalence of abdominal obesity (OR, 2.60; 95%CI, 1.31–5.17) in those who fasted for 10 to 12 h compared with those who fasted for less than 8 h. Among those who did not skip dinner, those who fasted for 10 to 12 h had an increased prevalence of elevated blood pressure (OR, 2.56; 95%CI, 1.35–4.85) compared with those who fasted for less than 8 h. Among those who did not skip dinner, fasting for 8 to 10 h decreased the prevalence of elevated triglycerides (OR, 0.41; 95%CI, 0.20–0.84) compared with that in those who fasted for less than 8 h.

## 4. Discussion

The results of this cross-sectional study indicated that adults consuming a higher number of meals daily (≥6 meals) have a lower prevalence of metabolic syndrome than those consuming a lower number of meals daily (≤3 meals) after adjusting for age, education, income, BMI, activity level, and smoking. After stratifying by gender, this association was true only among men. Furthermore, the risk of elevated triglycerides decreased in men as the frequency of meals increased, evidence of a dose–response relationship. The inverse association between meal frequency and elevated triglycerides was significant in adults consuming four and five meals daily but not in adults consuming six meals or more daily compared with those consuming three meals or less daily. This study found no significant association between fasting duration and metabolic syndrome; however, longer fasting duration was associated with an increased prevalence of elevated blood pressure and a decreased prevalence of elevated triglycerides.

In general, the study found that eating frequently was associated with a lower prevalence of metabolic syndrome and elevated triglycerides. Further, some associations were seen only in men and not women in the stratified analysis. These findings are consistent with those of previous studies that have reported an inverse association between meal frequency and metabolic syndrome [7]. In particular, the inverse association between meal frequency and abdominal obesity, elevated blood pressure, and elevated triglycerides was found only in Korean men [7]. Moreover, other studies have reported an inverse association between meal frequency and abdominal obesity in Swedish adults [20], and blood pressure in Korean adults [21]. A study has reported a reduced risk of triglycerides among those consuming more frequent meals [7]. On the other hand, other studies have reported opposing findings that a direct association exists between meal frequency and abdominal obesity in US adults [22], and BMI and waist circumference in British adults [23].

The inconsistency in the findings between metabolic syndrome, metabolic syndrome risk factors, and meal frequency could be due to a variety of factors. A possible reason for the discrepancy could be the differences in collecting dietary data or defining meal frequency, and the dietary assessment methods differ between the studies. Some studies have used 24 h dietary recall for dietary assessment [7,22], similar to the present study. Other studies have used a questionnaire for dietary assessment [20,21]. Furthermore, meal frequency differed between studies; some studies defined meals as any eating episode [7,20,22,23], while other studies defined meal frequency using only main meals and did not account for snacks [21]. In a culture where snacking is more prevalent and meal timing is more variable, it is difficult to distinguish between meals and snacks [24]. A study in Kuwait using the same data as those used in the present study reported that the population consumed 4.4 meals daily on average, defined as any eating episode, in a range of 1 meal to 10 meals [25].

In this study, fasting duration was not associated with metabolic syndrome. However, other studies have found an association between fasting duration and metabolic syndrome, including a recent study that found a significant inverse association between nightly fasting duration and metabolic syndrome in Iranian adults [26]. Moreover, in the present study, fasting duration was not always associated with a lower prevalence of metabolic syndrome risk factors. Among the study participants, longer fasting duration (>10 h) was associated with an increased prevalence of elevated blood pressure in adults after adjusting for age, education, income, BMI, activity level, and smoking. However, after stratifying by gender, this association was true only among women. Longer fasting duration (>8 h) was associated with a lower prevalence of elevated triglycerides in all adults after controlling for age, education, income, BMI, activity level, and smoking. After gender stratification, this association was true only in women for a longer fasting duration >12 h.

Few studies have been conducted on fasting duration and metabolic syndrome. The finding from the present study of an inverse association between longer fasting duration and the risk of elevated triglycerides aligns with findings from previous studies [7]. A study on Iranian adults that compared fasting durations of more than and less than 11.25 h found that longer fasting durations were significantly associated with a lower prevalence of elevated triglycerides [26]. While the present study found that longer fasting durations increased the prevalence of elevated blood pressure compared with shorter fasting durations, another study on prediabetic men reported that longer fasting durations improved blood pressure after five weeks of time-restricted feeding [27]. The differences in the demographics or health conditions of the populations could explain the discrepancy in the findings. Our study population consists mainly of healthy adults, while the study by Sutton et al. [27] was a population of prediabetic men.

To understand further whether or not fasting duration achieved by skipping dinner or skipping breakfast was associated differently with metabolic syndrome risk factors, the association of fasting duration with metabolic syndrome risk factors was assessed separately for those who skipped breakfast and skipped dinner. The study did not find an association between metabolic syndrome and fasting duration based on breakfast consumption. However, among those who skipped breakfast, an association was found of a reduced prevalence of elevated triglycerides for those who fasted more than 8 h compared with those who fasted for less than 8 h. Nonetheless, a recent cross-sectional study in Korea shows that eating in the morning is associated with a reduced risk of metabolic syndrome in adults, independent of meal frequency and fasting duration [7]. Moreover, a prospective study of adults aged 40–54 years found that eating at night leads to a higher prevalence of metabolic syndrome only in women and obesity in women and men [5]. This study’s findings do not show an association between eating at night and metabolic syndrome, but an increased prevalence of abdominal obesity and elevated blood pressure for those who eat at night and fast for 8 to 10 h. Further, an association of the reduced prevalence of metabolic syndrome was observed in our study with those who skipped dinner and fasted nightly for 8 to 10 h.

There are several limitations to this study. First, the study’s cross-sectional design makes it challenging to draw conclusions on the causal relationship between meal timing and frequency with metabolic syndrome and metabolic syndrome risk factors. Second, the study assessed diet using a 24 h dietary recall. Using 24 h dietary recall is a limitation because it captures diet on only one day and assumes that this is a representation of the individual’s daily diet. It is not the gold standard for assessment but is the most efficient and widely used dietary assessment method [28], especially for population-based studies. In fact, similar studies have used 24 h recall for dietary assessment [7]. Third, the low participation rate during the national survey introduced the potential for participation bias. In total, of the 1640 households that were contacted, only 545 households agreed to participate. This means that the KNNSS has a 33% response rate. Participation bias may have been introduced because those who agreed to participate may not have been a true representation of the overall population of Kuwait. Fourth, it was not possible to assess fasting duration >16 h, as a previous study reported the beneficial effects of longer fasting durations (>16 h) on cardiometabolic events in Korean adults [29]. Fifth, the data used in this study did not have information on sleep habits or patterns. Since sleep affects the circadian rhythm and the circadian rhythm affects metabolic syndrome risk factors [10], information on sleep could confound the relationship between meal timing and frequency and metabolic syndrome.

Nonetheless, this study has multiple strengths. First, even with the 33% response rate, the study used a nationally representative sample of healthy medication-free adults who were 20 years or older enrolled via a geographically representative method. Second, this study is the first to uncover the association between meal timing and frequency and metabolic syndrome in the Kuwaiti population. Third, the sufficient sample size of this study allowed for a stratified analysis of men and women separately.

## 5. Conclusions

In conclusion, eating more frequent meals or snacks may help decrease the prevalence of metabolic syndrome and elevated triglycerides in adults in Kuwait, particularly among men. No significant association was seen between fasting duration and metabolic syndrome; however, longer fasting duration was associated with a decrease in the prevalence of elevated triglycerides and an increase in the prevalence of elevated blood pressure. As the population of the study consists of Kuwaiti adults, the findings of this study may help in drafting recommendations and guidance for the general population. The generalizability of the study may be limited to medication-free adults; however, it is the first study to explore the association between meal timing and meal frequency. Given the limitations of this study, there is a need for further research to collect dietary data on multiple days and include information on sleep habits to further explore the relationship between meal timing and frequency and metabolic syndrome.

## Figures and Tables

**Table 1:** General characteristics of the study participants by gender.

	Total (*n* = 757)	Men (*n* = 341)	Women (*n* = 416)	*p*-Value ^a^
Age (years)	37.8 ± 12.3	38.6 ± 13.3	37.1 ± 11.3	0.10
Education, *n* (%)				0.22
Highschool or below	323 (42.7)	155 (45.5)	168 (40.4)	
College graduate	403 (53.2)	170 (49.9)	233 (56.0)	
Higher education	31 (4.1)	16 (4.7)	15 (3.6)	
Household income				<0.001
Low	480 (63.7)	152 (45.0)	328 (78.9)	
Middle	222 (29.4)	147 (43.5)	75 (18.0)	
High	52 (6.9)	39 (11.5)	13 (3.1)	
Physically activity				<0.001
Active	310 (41)	180 (52.8)	130 (31.3)	
Sedentary	446 (59)	161 (47.2)	285 (68.7)	
Smoking (ever)	251 (33.29)	211 (62.4)	40 (9.6)	<0.001
BMI (kg/m^2^)	29.2 ± 6.4	28.3 ± 5.9	29.9 ± 6.7	<0.001
Waist circumference (cm)	93.3 ± 16.3	98.9 ± 14.7	88.6 ± 16.1	<0.001
Systolic blood pressure (mmHg)	124.9 ± 17.1	129.1 ± 16.8	121.5 ± 16.7	<0.001
Diastolic blood pressure (mmHg)	80.5 ± 11.4	82.7 ± 10.9	78.6 ± 11.3	<0.001
HDL cholesterol (mmol/L)	1.2 ± 0.3	1.1 ± 0.2	1.3 ± 0.3	<0.001
Triglycerides (mmol/L)	1.3 ± 0.74	1.4 ± 0.8	1.1 ± 0.6	<0.001
Fasting blood glucose (mmol/L)	5.6 ± 1.6	5.7 ± 1.9	5.4 ± 1.3	0.02
HbA1c	5.7 ± 0.9	5.8 ± 1.1	5.6 ± 0.7	0.02
Total energy intake (kcal/day)	2063.9 ± 967.0	2491.8 ± 1034.7	1712.8 ± 743.1	<0.001
Meal frequency	4.4 ± 1.0	4.5 ± 1.1	4.3 ± 1.0	<0.001
Nightly fasting duration (hours)	9.8 ± 3.3	10.1 ± 2.8	10.9 ± 3.1	<0.001
Skipped breakfast	227 (30.0)	80 (10.6)	147 (19.4)	<0.001
Skipped dinner	74 (9.8)	33 (4.4)	41 (5.4)	0.93
Late night dinner	408 (53.9)	198 (26.2)	210 (27.7)	0.04
Abdominal obesity	360 (40.5)	208 (61.0)	283 (63.0)	0.04
Prediabetes	226 (29.9)	114 (33.4)	112 (26.9)	0.05
Elevated triglycerides	172 (22.7)	102 (29.9)	70 (16.8)	<0.001
Low HDL cholesterol	306 (56.5)	105 (30.8)	201 (48.3)	<0.001
Elevated blood pressure	351 (46.4)	194 (56.9)	157 (37.7)	<0.001
Metabolic syndrome	261 (34.5)	124 (36.4)	137 (32.9)	0.32
Risk factors for metabolic syndrome				
None	95 (12.6)	45 (13.2)	50 (12.6)	0.63
One	171 (22.6)	61 (17.9)	110 (26.4)	0.005
Two	230 (30.4)	111 (32.6)	119 (28.6)	0.24
Three	151 (20.0)	66 (19.4)	85 (20.4)	0.71
Four	88 (11.6)	48 (14.1)	40 (9.6)	0.06
Five	22 (2.9)	10 (2.9)	12 (2.9)	0.97

^a^ *p*-value for *t*-test and Chi-square test.

**Table 2:** Multivariable-adjusted odds ratios and 95% CL for metabolic syndrome according to meal frequency and fasting duration for all participants.

	Metabolic Syndrome	Abdominal Obesity	Elevated Blood Pressure	Low HDL Cholesterol	Elevated Triglycerides	Elevated Fasting Blood Glucose
Meal Frequency						
Three meals (*n* = 190)	1.0	1.0	1.0	1.0	1.0	1.0
Four meals (*n* = 226)	0.67 (0.34–1.33)	0.89 (0.44–1.81)	0.54 (0.31–0.94)	1.00 (0.57–1.76)	0.69 (0.34–1.41)	0.90 (0.47–1.72)
Five meals (*n* = 208)	0.78 (0.37–1.66)	1.04 (0.51–2.11)	1.06 (0.59–1.91)	1.01 (0.55–1.84)	0.68 (0.32–1.45)	0.84 (0.42–1.70)
Six meals (*n* = 133)	0.43 (0.19–0.96)	0.73 (0.28–1.91)	0.59 (0.30–1.13)	1.17 (0.59–2.33)	0.56 (0.23–1.39)	0.61 (0.27–1.38)
*p*-value for trend	0.09	0.63	0.49	0.70	0.22	0.22
Fasting duration						
<8 h (*n* = 156)	1.0	1.0	1.0	1.0	1.0	1.0
8–10 h (*n* = 229)	0.83 (0.44–1.58)	1.99 (0.90–4.38)	1.51 (0.82–2.75)	0.89 (0.48–1.67)	0.47 (0.24–0.91)	1.04 (0.55–1.98)
10–12 h (*n* = 196)	1.60 (0.76–3.38)	2.10 (1.00–4.44)	2.26 (1.22–4.20)	0.61 (0.32–1.15)	0.58 (0.28–1.22)	1.90 (0.93–3.87)
>14 h (*n* = 176)	1.85 (0.86–3.98)	2.16 (0.99–4.71)	1.91 (1.01–3.33)	0.85 (0.44–1.66)	0.56 (0.25–1.26)	1.87 (0.88–3.95)
*p*-value for trend	0.03	0.10	0.02	0.39	0.36	0.03

Note: Adjusted for age (years), education, income, BMI, activity level, and smoking.

**Table 3:** Multivariable-adjusted odds ratio and 95% CL for metabolic syndrome according to meal frequency and fasting duration for men.

	Metabolic Syndrome	Abdominal Obesity	Elevated Blood Pressure	Low HDL Cholesterol	Elevated Triglycerides	Elevated Fasting Blood Glucose
Meal Frequency						
Three meals (*n* = 71)	1.0	1.0	1.0	1.0	1.0	1.0
Four Meals (*n* = 96)	0.51 (0.17–1.50)	0.97 (0.28–3.44)	0.73 (0.32–1.67)	0.52 (0.19–1.43)	0.31 (0.12–0.82)	0.82 (0.31–2.13)
Five Meals (*n* = 99)	0.45 (0.16–1.28)	1.30 (0.33–5.04)	0.66 (0.28–1.54)	0.69 (0.26–1.86)	0.23 (0.09–0.60)	1.24 (0.43–3.61)
Six meals (*n* = 75)	0.26 (0.09–0.76)	0.35 (0.08–1.52)	0.47 (0.19–1.19)	0.51 (0.18–1.47)	0.32 (0.10–1.01)	0.94 (0.32–2.70)
*p* for trend	0.03	0.21	0.17	0.36	0.04	0.74
Fasting duration						
<8 h (*n* = 86)	1.0	1.0	1.0	1.0	1.0	1.0
8–10 h (*n* = 116)	0.69 (0.29–1.64)	2.00 (0.64–6.30)	1.14 (0.49–2.68)	0.84 (0.35–2.03)	0.53 (0.22–1.27)	1.14 (0.49–2.67)
10–12 h (*n* = 76)	1.18 (0.47–2.94)	2.16 (0.70–6.70)	1.42 (0.58–3.45)	0.56 (0.22–1.42)	0.49 (0.18–1.30)	1.84 (0.69–4.91)
>12 h (*n* = 63)	3.66 (0.75–17.85)	2.40 (0.69–8.40)	2.38 (0.88–6.43)	1.38 (0.41–4.59)	1.52 (0.34–6.77)	2.03 (0.62–6.59)
*p* for trend	0.04	0.18	0.07	0.92	0.66	0.16

Note: Adjusted for age (years), education, income, BMI, activity level, and smoking.

**Table 4:** Multivariable-adjusted odds ratio and 95% CL for metabolic syndrome according to meal frequency and fasting duration for women.

	Metabolic Syndrome	Abdominal Obesity	Elevated Blood Pressure	Low HDL Cholesterol	Elevated Triglycerides	Elevated Fasting Blood Glucose
Meal Frequency						
Three meals (*n* = 119)	1.0	1.0	1.0	1.0	1.0	1.0
Four Meals (*n* = 130)	0.77 (0.29–2.00)	1.15 (0.47–2.80)	0.39 (0.18–0.86)	1.31 (0.64–2.68)	1.28 (0.51–3.25)	1.01 (0.41–2.46)
Five Meals (*n* = 109)	1.17 (0.41–3.37)	0.92 (0.39–2.20)	1.48 (0.62–3.54)	1.23 (0.54–2.77)	2.08 (0.54–7.95)	0.66 (0.25–1.78)
Six meals (*n* = 58)	0.62 (0.19–2.08)	1.35 (0.32–5.64)	0.60 (0.23–1.56)	1.94 (0.70–5.44)	0.72 (0.18–2.93)	0.35 (0.12–1.04)
*p* for trend	0.72	0.75	0.85	0.25	0.97	0.04
Fasting duration						
<8 h (*n* = 70)	1.0	1.0	1.0	1.0	1.0	1.0
8–10 h (*n* = 113)	1.12 (0.43–2.94)	2.08 (0.63–6.83)	2.17 (0.87–5.41)	0.91 (0.37–2.24)	0.33 (0.09–1.24)	0.91 (0.36–2.32)
10–12 h (*n* = 120)	1.95 (0.56–6.73)	1.65 (0.55–4.93)	2.88 (1.15–7.20)	0.74 (0.30–1.84)	0.44 (0.10–2.03)	1.90 (0.63–5.77)
>12 h (*n* = 113)	1.39 (0.48–4.04)	2.07 (0.77–5.56)	1.80 (0.75–4.29)	0.82 (0.33–2.05)	0.19 (0.06–0.63)	1.60 (0.56–4.54)
*p* for trend	0.37	0.36	0.20	0.58	0.02	0.18

Note: Adjusted for age (years), education, income, BMI, activity level, and smoking.

**Table 5:** Multivariable-adjusted odds ratios and 95% CL for metabolic syndrome according to meal frequency and fasting duration for women defined by breakfast consumption.

Fasting Duration	Metabolic Syndrome	Abdominal Obesity	Elevated Blood Pressure	Low HDL Cholesterol	Elevated Triglycerides	Elevated Fasting Blood Glucose
Skip breakfast						
<8 h (*n* = 113)	1.0	1.0	1.0	1.0	1.0	1.0
8–10 h (*n* = 210)	0.32 (0.03–3.21)	1.58 (0.16–15.69)	0.62 (0.11–3.31)	0.76 (0.13–4.36)	0.04 (0.006–0.25)	0.96 (0.14–6.56)
10–12 h (*n* = 144)	0.72 (0.17–3.00)	1.83 (0.52–6.40)	1.78 (0.54–5.80)	0.39 (0.10–1.45)	0.15 (0.03–0.90)	1.03 (0.20–5.24)
>12 h (*n* = 63)	0.91 (0.25–3.29)	1.13 (0.38–3.56)	0.79 (0.29–2.19)	1.07 (0.34–3.33)	0.14 (0.04–0.48)	1.63 (0.43–6.28)
*p* for trend	0.87	0.90	0.76	0.97	0.28	0.34
Did not skip breakfast						
<8 h (*n* = 43)	1.0	1.0	1.0	1.0	1.0	1.0
8–10 h (*n* = 19)	0.84 (0.42–1.67)	1.38 (0.65–2.93)	1.79 (0.87–3.68)	0.74 (0.37–1.49)	0.49 (0.24–1.02)	0.82 (0.39–1.70)
10–12 h (*n* = 52)	1.56 (0.73–3.33)	2.21 (1.04–4.71)	2.23 (1.06–4.71)	0.67 (0.32–1.44)	0.51 (0.22–1.21)	1.62 (0.70–3.74)
>12 h (*n* = 113)	0.98 (0.34–2.83)	1.05 (0.37–2.94)	3.31 (1.27–8.59)	0.47 (0.19–1.16)	0.46 (0.11–2.02)	0.89 (0.30–2.60)
*p* for trend	0.39	0.30	0.009	0.19	0.31	0.41

Note: Adjusted for age (years), gender, BMI, activity level, and smoking.

**Table 6:** Multivariable-adjusted odds ratio and 95% CL for metabolic syndrome according to meal frequency and fasting duration defined by dinner consumption.

Fasting Duration	Metabolic Syndrome	Abdominal Obesity	Elevated Blood Pressure	Low HDL Cholesterol	Elevated Triglycerides	Elevated Fasting Blood Glucose
Skip dinner						
<8 h (*n* = 137)	1.0	1.0	1.0	1.0	1.0	1.0
8–10 h (*n* = 214)	0.06 (0.007–0.49)	0.02 (0.001–0.43)	0.18 (0.02–1.61)	1.06 (0.18–6.23)	0.41 (0.04–3.68)	0.55 (0.07–4.34)
10–12 h (*n* = 189)	0.19 (0.006–6.62)	0.04 (0.001–2.46)	1.52 (0.13–17.89)	0.29 (0.02–3.96)	0.19 (0.02–2.15)	1.08 (0.03–35.58)
>12 h (*n* = 143)	0.24 (0.04–1.47)	0.31 (0.04–2.28)	1.41 (0.18–11.22)	0.38 (0.05–2.71)	0.42 (0.02–8.33)	1.59 (0.17–15.24)
*p* for trend	0.56	0.19	0.34	0.20	0.34	0.54
Do not skip dinner						
<8 h (*n* = 19)	1.0	1.0	1.0	1.0	1.0	1.0
8–10 h (*n* = 15)	0.99 (0.51–1.94)	1.76 (0.85–3.65)	1.90 (1.00–3.59)	0.91 (0.47–1.77)	0.41 (0.20–0.84)	0.89 (0.45–1.76)
10–12 h (*n* = 7)	1.81 (0.90–3.61)	2.60 (1.31–5.17)	2.56 (1.35–4.85)	0.81 (0.42–1.57)	0.56 (0.26–1.22)	1.76 (0.84–3.66)
>12 h (*n* = 33)	1.30 (0.60–2.80)	1.34 (0.64–2.82)	1.57 (0.76–3.17)	0.91 (0.45–1.84)	0.43 (0.17–1.06)	1.48 (0.64–3.42)
*p* for trend	0.20	0.29	0.16	0.70	0.16	0.11

Note: Adjusted for age (years), gender, BMI, activity level, and smoking.

## Data Availability

The data presented in this study are available on request from the corresponding author due to privacy and ethical restrictions.

## References

[1] Grundy S.M. (2008). Metabolic syndrome pandemic. Arterioscler. Thromb. Vasc. Biol..

[2] Li X., Li X., Lin H., Fu X., Lin W., Li M., Zeng X., Gao Q. (2017). Metabolic syndrome and stroke: A meta-analysis of prospective cohort studies. J. Clin. Neurosci..

[3] Saboya P.P., Bodanese L.C., Zimmermann P.R., Gustavo A.D.S., Assumpção C.M., Londero F. (2016). Metabolic syndrome and quality of life: A systematic review. Rev. Lat.-Am. Enferm..

[4] Boudreau D., Malone D., Raebel M., Fishman P.A., Nichols G.A., Feldstein A.C., Boscoe A.N., Ben-Joseph R.H., Magid D.J., Okamoto L.J. (2009). Health care utilization and costs by metabolic syndrome risk factors. Metab. Syndr. Relat. Disord..

[5] Yoshida J., Eguchi E., Nagaoka K., Ito T., Ogino K. (2018). Association of night eating habits with metabolic syndrome and its components: A longitudinal study. BMC Public Health.

[6] Alkhulaifi F., Darkoh C. (2022). Meal Timing, Meal Frequency and Metabolic Syndrome. Nutrients.

[7] Ha K., Song Y. (2019). Associations of meal timing and frequency with obesity and metabolic syndrome among Korean adults. Nutrients.

[8] Jiang P., Turek F.W. (2017). Timing of meals: When is as critical as what and how much. Am. J. Physiol.-Endocrinol. Metab..

[9] Teixeira G.P., Guimarães K.C., Soares A.G.N., Marqueze E.C., Moreno C.R., Mota M.C., Crispim C.A. (2023). Role of chronotype in dietary intake, meal timing, and obesity: A systematic review. Nutr. Rev..

[10] Zimmet P., Alberti K.G., Stern N., Bilu C., El-Osta A., Einat H., Kronfeld-Schor N. (2019). The Circadian Syndrome: Is the Metabolic Syndrome and much more!. J. Intern. Med..

[11] St-Onge M.P., Ard J., Baskin M.L., Chiuve S.E., Johnson H.M., Kris-Etherton P., Varady K. (2017). Meal timing and frequency: Implications for cardiovascular disease prevention: A scientific statement from the American Heart Association. Circulation.

[12] Wang X., Hu Y., Qin L.Q., Dong J.Y. (2022). Meal frequency and incidence of type 2 diabetes: A prospective study. Br. J. Nutr..

[13] Titan S.M., Bingham S., Welch A., Luben R., Oakes S., Day N., Khaw K.T. (2001). Frequency of eating and concentrations of serum cholesterol in the Norfolk population of the European prospective investigation into cancer (EPIC-Norfolk): Cross sectional study. BMJ.

[14] Xiao Q., Bauer C., Layne T., Playdon M. (2021). The association between overnight fasting and body mass index in older adults: The interaction between duration and timing. Int. J. Obes..

[15] Noubiap J.J., Nansseu J.R., Lontchi-Yimagou E., Nkeck J.R., Nyaga U.F., Ngouo A.T., Tounouga D.N., Tianyi F.L., Foka A.J., Ndoadoumgue A.L. (2022). Geographic distribution of metabolic syndrome and its components in the general adult population: A meta-analysis of global data from 28 million individuals. Diabetes Res. Clin. Pract..

[16] Shin S., Jee H. (2020). Prevalence of metabolic syndrome in the Gulf Cooperation Council countries: Meta-analysis of cross-sectional studies. J. Exerc. Rehabil..

[17] Al Zenki S., Al Omirah H., Al Hooti S., Al Hamad N., Jackson R.T., Rao A., Al Jahmah N., Al Obaid I.A., Al Ghanim J., Al Somaie M. (2012). High prevalence of metabolic syndrome among Kuwaiti adults—A wake-up call for public health intervention. Int. J. Environ. Res. Public Health.

[18] George Alberti P.Z., Shaw J. (2006). The IDF Consensus Worldwide Definition of the Metabolic Syndrome.

[19] SAS (2013). SAS.

[20] Holmbäck I., Ericson U., Gullberg B., Wirfält E. (2010). A high eating frequency is associated with an overall healthy lifestyle in middle-aged men and women and reduced likelihood of general and central obesity in men. Br. J. Nutr..

[21] Kim S., Park G.H., Yang J.H., Chun S.H., Yoon H.J., Park M.S. (2014). Eating frequency is inversely associated with blood pressure and hypertension in Korean adults: Analysis of the Third Korean National Health and Nutrition Examination Survey. Eur. J. Clin. Nutr..

[22] Murakami K., Livingstone M.B.E. (2015). Eating frequency is positively associated with overweight and central obesity in US adults. J. Nutr..

[23] Murakami K., Livingstone M. (2014). Eating frequency in relation to body mass index and waist circumference in British adults. Int. J. Obes..

[24] Kant A., Graubard B. (2015). 40-year trends in meal and snack eating behaviors of American adults. J. Acad. Nutr. Diet.

[25] Alkhulaifi F., Al-Hooti S., Al-Zenki S., AlOmirah H., Darkoh C. (2023). Dietary Habits, Meal Timing, and Meal Frequency in Kuwaiti Adults: Analysis of the Kuwait National Nutrition Surveillance Data. Nutrients.

[26] Zeraattalab-Motlagh S., Lesani A., Janbozorgi N., Djafarian K., Majdi M., Shab-Bidar S. (2023). Association of nightly fasting duration, meal timing and frequency with the metabolic syndrome among Iranian adults. Br. J. Nutr..

[27] Sutton E.F., Beyl R., Early K.S., Cefalu W.T., Ravussin E., Peterson C.M. (2018). Early time-restricted feeding improves insulin sensitivity, blood pressure, and oxidative stress even without weight loss in men with prediabetes. Cell Metab..

[28] Dao M.C., Subar A.F., Warthon-Medina M., Cade J.E., Burrows T., Golley R.K., Forouhi N.G., Pearce M., Holmes B.A. (2019). Dietary assessment toolkits: An overview. Public Health Nutr..

[29] Makarem N., Sears D.D., St-Onge M.P., Zuraikat F.M., Gallo L.C., Talavera G.A., Castaneda S.F., Lai Y., Mi J., Aggarwal B. (2020). Habitual nightly fasting duration, eating timing, and eating frequency are associated with cardiometabolic risk in women. Nutrients.

